# Comparison between intravenous chemotherapy and intra-arterial chemotherapy for retinoblastoma: a meta-analysis

**DOI:** 10.1186/s12885-018-4406-6

**Published:** 2018-04-27

**Authors:** Qiuying Chen, Bin Zhang, Yuhao Dong, Xiaokai Mo, Lu Zhang, Wenhui Huang, Hua Jiang, Jiejun Xia, Shuixing Zhang

**Affiliations:** 1 Department of Radiology, the First Affiliated Hospital, Jinan University, No.613, Huangpu West Road, Tianhe District, Guangzhou, Guangdong 510627 People’s Republic of China; 2 Graduate College, Jinan University, Guangzhou, Guangdong People’s Republic of China; 3 Department of Radiology, Guangdong General Hospital/Guangdong Academy of Medical Sciences, Guangzhou, Guangdong People’s Republic of China; 4 Graduate College, Southern Medical University, Guangzhou, Guangdong People’s Republic of China; 5 School of medicine, South China University of Technology, Guangzhou, Guangdong People’s Republic of China; 6 Department of Interventional Radiology and Vascular Anomalies, Women’s and Children Medical Center, Guangzhou, China

**Keywords:** Retinoblastoma, Intravenous chemotherapy, Intra-arterial chemotherapy, Meta-analysis

## Abstract

**Background:**

Intravenous chemotherapy (IVC) and intra-arterial chemotherapy (IAC) have become the primary treatments for retinoblastoma; however, some controversy remains over which method is more effective. We conducted a meta-analysis to compare the clinical efficacy of IVC and IAC.

**Methods:**

We systematically searched literature published on PubMed, Embase, and Cochrane Library up to May 2017. Studies containing either IAC or IVC that reported on efficacy were included. The effects estimate was expressed as a pooled rate with 95% confidence interval (CI), using a fixed-effects or random-effects model.

**Results:**

Twenty-six studies were identified which included 1541 eyes (IAC: 11 trials, 445 eyes; IVC: 16 trials, 1096 eyes). The mean follow-up times were 49.4 months (range, 13.0–105.3 months) for IVC and 21.7 months (range, 8.8–38.7 months) for IAC. For the International Classification of Intraocular Retinoblastoma (ICRB) grading, the overall success rate was higher with IAC than with IVC (75.7% [95%CI: 65.7%–83.6%] vs. 69.5% [95%CI: 51.9%–82.8%], *P* < 0.001). The globe salvage with IAC was higher than with IVC in group D eyes (79.5% [95%CI: 71.8%–85.4%] vs. 55.1% [95%CI: 45.6%–64.2%], P < 0.001), but not in groups B (95.8% [95%CI: 57.5%–99.7%] vs. 82.5% [95%CI: 58.9%–94.0%], *P* = 0.163), C (91.3% [95%CI: 65.9%–98.3%] vs. 89.0% [95%CI: 69.0%–96.7%], *P* = 0.212), and E eyes (51.2% [95%CI: 37.0%–65.2%] vs. 43.2% [95%CI: 18.3%–72.1%], *P* = 0.578). IAC and IVC were not significantly different regarding the recurrence and metastasis rates (15.0% vs. 15.4%, *P* = 0.148 and 2.7% vs. 0.6%, *P* = 0.194, respectively). For Reese-Ellsworth (RE) grading, IAC had a higher globe salvage in groups IV (90.9% [95%CI: 56.0%–98.7%] vs. 66.3% [95%CI: 32.4%–89.0%], *P* = 0.047) and V eyes (83.2% [95%CI: 72.0%–90.5%] vs. 59.9% [95%CI: 43.1%–74.6%], *P* = 0.003), but not in group I-III eyes (88.6% [95%CI: 58.3%–97.7%] vs. 88.1% [95%CI: 76.6%–94.4%], *P* = 0.244). The overall success rate was higher in IAC than in IVC (87.1% [95%CI: 78.1%–92.7%] vs. 77.3% [95%CI: 68.1%–84.4%], *P* = 0.033).

**Conclusions:**

IAC may be superior to IVC for the treatment of retinoblastoma, with a higher overall success rate and higher globe salvage in group D or groups IV and V eyes.

**Electronic supplementary material:**

The online version of this article (10.1186/s12885-018-4406-6) contains supplementary material, which is available to authorized users.

## Background

Retinoblastoma is the most common intraocular malignancy of childhood (approximately 1/15,000–20,000 live births) and it accounts for 4% of all pediatric cancers [1]. Two-thirds of all cases of retinoblastoma are diagnosed before the age of two years. All patients with bilateral retinoblastoma and approximately 10%–15% of children with unilateral disease carry a germline mutation which is transmissible to their off spring [2]. In developed countries, the survival rate approaches 98%; however, due to the limitations of health care in low-income countries, it is much lower at 40% [3, 4]. Before the 1990s, retinoblastoma was mainly treated using enucleation and external beam radiotherapy (EBRT). However, these methods are associated with numerous complications, including loss of vision and severe toxic side effects. Currently, first-line conservative management of retinoblastoma has moved from EBRT and enucleation to intravenous chemotherapy (IVC) or intra-arterial chemotherapy (IAC), consolidated by focal treatment. The therapeutic efficacy of IVC and IAC has received increasing attention.

IVC was first used in 1953 [5]. The standard protocol is the vincristine-etoposide-carboplatin (VEC) program used as a combination triple-drug therapy, typically performed for six cycles [6]. VEC may yield better results in eyes with advanced cancer when combined with local therapeutic methods. The detrimental effect of IVC on children is worth noting. Since eyes with vitreous or subretinal seeding are less sensitive to IVC treatment, IVC may not perform well in cases of advanced tumors. In addition, IVC is associated with certain adverse reactions: carboplatin is known to cause ototoxicity and etoposide has the potential to cause acute lymphoblastic leukemia.

**Fig. 1:**
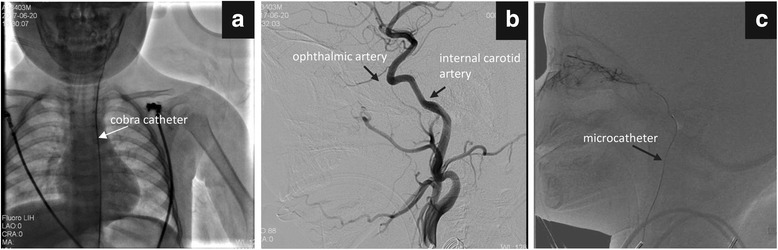
The technique of intra-arterial chemotherapy (IAC) treatment. **a**: An arteriogram was performed to indicate the takeoff of the ophthalmic artery from the internal carotid artery. **b**: Using fluoroscopy and roadmap guidance, the microcatheter selectively catheterized the ophthalmic artery. **c**: As soon as the microcatheter was in a stable position at the ostium of the ophthalmic artery, then pulse-inject drugs

IAC was first described by Reese and colleagues in 1950 [7]. The technique was pioneered in Japan [8] and was later popularized by Abramson et al. [9]. The procedure involves directly injecting concentrated doses of chemotherapeutic drugs (melphalan, topotecan, or carboplatin) into the ophthalmic artery using a modern microcatheter (Fig. 1), to increase the concentration of chemotherapy drugs 10- to 30-fold at the tumor site. Consequently, the concentration of the drugs in the peripheral blood is minimal. Each eye requires an average of three treatment cycles and each cycle is planned at a 4-week interval. Successful treatment is indicated by a decrease in the size of the tumor. Remaining tumors can be eliminated using laser, cryotherapy, or radioactive plaques. Previous studies have reported the efficacy and safety of this approach [10–13]. However, due to the high concentration of the chemical drugs that are used in IAC, local complications are very high. Thus, the issues around improving globe salvage as well as reducing the risk of local complications must be addressed urgently.

At present, both IVC and IAC are first-line treatments for retinoblastoma in the clinical setting. Although IAC has been shown to have excellent therapeutic effects, whether it can replace IVC remains controversial. Some single-arm studies have reported either on IAC or IVC, but only one study compared these two methods in unilateral group D retinoblastoma [14]. Moreover, the outcomes of studies that evaluated success rates of these methods have been discrepant due to the varying quality of the research and the different sources and capacities of the samples. Hence, we performed this study to compare the clinical use of IAC and IVC to identify which method is more effective or to determine if both are necessary for the treatment of retinoblastoma. The improved quality of evidence resulting from this meta-analysis is expected to be helpful for doctors in their clinical practice.

## Methods

### Search strategy

PubMed, Embase, and Cochrane Library databases were searched without geographical, publication type, and language restrictions for literature published up to May 2017. The literature was searched by combining Medical Subject Headings (Mesh) and multiple free words. Mesh and free words were combined using a logical ‘OR’ operator. The results of the Mesh and free words search were combined using a logical ‘AND’ operator. As the search terms and strategy were not perfect, we also used references from papers to supplement the results. The search strings used in PubMed were as follows:

#1: (retinoblastoma[mesh]) OR (retinoblastomas) OR (neuroblastoma, retinal) OR (neuroblastomas, retinal) OR (retinalneuroblastoma) OR (retinal neuroblastomas) OR (glioma, retinal) OR (gliomas, retinal) OR (retinal glioma) OR (retinal gliomas) OR (eye cancer, retinoblastoma) OR (glioblastoma, retinal) OR (glioblastomas, retinal) OR (retinal glioblastoma) OR (retinal glioblastomas) OR (sporadic retinoblastoma) OR (retinoblastoma, sporadic) OR (retinoblastomas, sporadic) OR (sporadic retinoblastomas) OR (familial retinoblastoma) OR (familial retinoblastomas) OR (retinoblastoma, familial) OR (retinoblastomas, familial) OR (hereditary retinoblastoma) OR (hereditary retinoblastomas) OR (retinoblastoma, hereditary) OR (retinoblastomas, hereditary),

#2: (drug therapy [mesh]) OR (drug therapies) OR (therapies, drug) OR (chemotherapy) OR (chemotherapies) OR (pharmacotherapies) OR (therapy, drug) OR (pharmacotherapy),

#3: #1 AND #2,

#4: (humans [mesh]) NOT (animals [mesh]),

#5: #3 AND #4.

### Inclusion criteria

Two authors (LZ and YHD) reviewed the articles by reading the title, abstract, and full text independently; a third author (XKM) resolved disagreements and recorded the excluded study as well as the reasons for its exclusion. Inclusion criteria were as follows: (1) only IAC or IVC as the primary treatment method (not used in combination); (2) eyes were grouped according to the International Classification of Intraocular Retinoblastoma (ICRB) system, or the Reese-Ellsworth (RE) classification system; (3) studies that provided efficacy data; (4) more than 10 eyes included in a study; and (5) when we encountered multiple publications of the same clinical trial, we selected the latest or most complete publication. We excluded studies if they were published as reviews or case reports.

### Classification of retinoblastoma

**Fig. 2:**
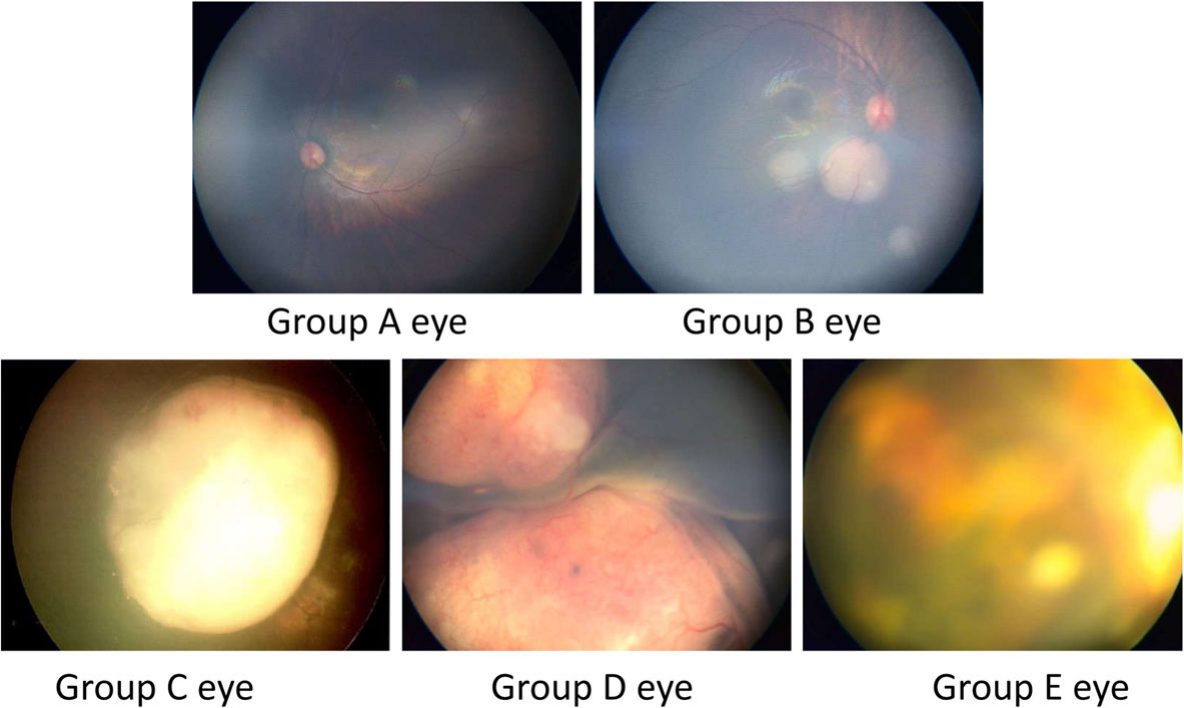
The fundus photographs of group A-E eyes of retinoblastoma. **Group A:** Eyes with small discrete tumors away from critical structures; **Group B:** Eyes with no vitreous or subretinal seeding and discrete retinal tumor of any size or location; **Group C:** Eyes with only focal vitreous or subretinal seeding and discrete retinal tumors of any size and location; **Group D:** Eyes with diffuse vitreous or subretinal seeding and/or massive, nondiscrete endophytic or exophytic disease; **Group E:** Eyes that have been destroyed anatomically or functionally by the tumor

Classification of retinoblastoma is necessary to formulate appropriate management strategies and improve predictability of the treatment outcomes. Formerly, RE grading was widely used; however, it was later replaced by ICRB system which included the Philadelphia version and the Children’s Hospital Los Angeles (CHLA) version [15]. Therefore, RE grading are mainly reported in earlier articles while ICRB system are mainly reported in articles published in recent years. In order to include valuable data as much as possible, we selected RE grading and ICRB system as the basis for grouping. Fig. 2 shows the fundus photographs of groups A to E eyes.

### Data synthesis and analysis

Meta-analysis was performed for an outcome only if there were appropriate data for at least two single arms in each group. The rates of different groups were used as weighting variables and expressed as a pooled rate with 95% confidence intervals (CI). We evaluated heterogeneity across studies using I^2^ statistics, with values of > 25%, > 50%, and > 75% representing mild, moderate, and grievous heterogeneity, respectively. If I^2^ > 50%, a random-effects model was applied in case of statistical heterogeneity, otherwise a fixed-effects model was used. We used the Chi-square test or Fisher’s exact test to compare the rates of different groups. A *P*-value of < 0.05 was considered statistically significant. Statistical analyses were performed using Comprehensive Meta-Analysis version 2 and SPSS version 16.

## Results

### Study characteristics

**Fig. 3:**
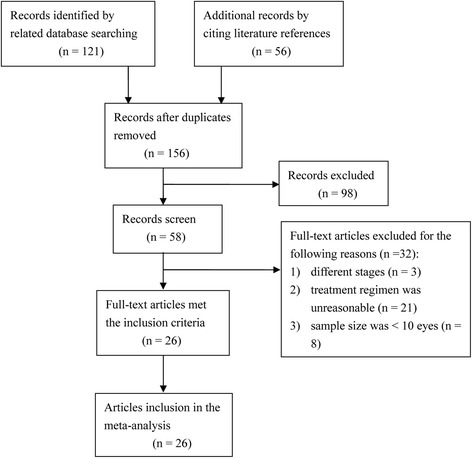
The literature screening flowchart

One hundred and fifty-six records were selected by searching the databases and citing literature references. After 130 exclusions, 26 articles qualified for the meta-analysis (Fig. 3). The trials included 1402 eyes that received either IAC (11 trials, 445 eyes) or IVC (16 trials, 1096 eyes) as the primary treatment modality up to May 2017. The protocol of IAC was ophthalmic artery chemotherapy infusion under fluoroscopic guidance using melphalan in every case, with additional topotecan and/or carboplatin as necessary (ten trials). The dose was determined by the patient’s age. The protocols of IVC included: six-cycle VEC (13 trials), 13-cycle VEC (one trial), six-cycle vincristine-carboplatin (one trial), and six-cycle etoposide-carboplatin (one trial) accompanied by local consolidation therapy (cryotherapy, photocoagulation, thermotherapy, and brachytherapy). Twenty-one studies [11, 14, 16–32] reported ICRB grading and seven studies [11, 32–37] reported RE grading, two of which reported two gradings. The mean follow-up time was 49.4 months in IVC and 21.7 months in IAC. Details of eyes in each enrolled trial are listed in Tables 1, 2, and 3. The primary endpoints included globe salvage in each group of eyes and overall success rate. Overall success was defined as avoidance of EBRT or enucleation. The secondary endpoints were recurrence rate, the occurrence of adverse events, tumor metastasis rate, and second malignant neoplasms (SMNs) incidence. The outcomes of the meta-analysis are shown in Additional files 1, 2, 3, and 4.

**Table 1:** Details of trials included in IVC treatment based on ICRB grading

Authors	Mean follow -up (months)	Globe salvage in different group eyes (%)					Overall Success Rate (%)	Second Tumor Rate (%)	Metastasis Rate (%)	Recurrence Rate (%)
		A (*n* = 31)	B (*n* = 142)	C (*n* = 23)	D (*n* = 458)	E (*n* = 106)				
Shields2006	NA	100.0(23/23)	92.7(89/96)	90.5(19/21)	46.8(51/109)	NA	NA	NA	NA	NA
Shin2010	53.3	100.0(8/8)	71.4(10/14)	NA	35.7(15/42)	100.0(1/1)	52.3(34/65)	NA	NA	NA
Kaliki2011	66.0	NA	NA	NA	NA	NA	NA	0.0(0/52)	0.0(0/52)	7.7 (4/52)
Turaka2011	80.0	NA	NA	NA	NA	NA	NA	3.8(6/156)	NA	NA
Berry2013	45.4	NA	NA	NA	47.3(26/55)	NA	NA	0.0(0/55)	0.0(0/55)	53.7 (29/55)
Cohen2009	30.0	NA	NA	NA	61.1(11/18)	NA	NA	0.0(0/18)	NA	NA
Berry2017(1)	86.3	NA	NA	NA	70.6(72/102)	24.3(9/37)	58.3(81/139)	NA	0.7(1/139)	0.7(1/139)-
Fabian2017	64.4	NA	NA	NA	62.5(40/64)	NA	NA	NA	0.0(0/64)	NA
Friedman2017	24.0	NA	64.0(16/25)	NA	NA	NA	NA	NA	0.0(0/25)	NA
Shields2009	24.0	NA	NA	NA	NA	59.1(39/66)	NA	1.6 (1/64)	NA	NA
Yousef2017	NA	NA	100.0(7/7)	100.0(2/2)	75.0(12/16)	0.0(0/2)	83.3(25/30)	0.0(0/30)	NA	NA
Berry2017(2)	33.0	NA	NA	NA	48.1(25/52)	NA	NA	NA	NA	9.6 (5/52)
Munier2017	105.3	NA	NA	NA	NA	NA	NA	NA	NA	52.2 (12/23)

Note: Globe salvage: avoidance of enucleation or EBRT. Abbreviations: IVC = intravenous chemotherapy; NA = not applicable

**Table 2:** Details of trials included in IAC treatment based on ICRB grading

Authors	Mean follow -up (months)	Globe salvage in different group eyes (%)					Overall Success Rate (%)	Second Tumor Rate (%)	Metastasis Rate (%)	Recurrence Rate (%)	Secondary treatment success rate (%)
		A (*n* = 0)	B (*n* = 12)	C (*n* = 17)	D (*n* = 148)	E (*n* = 49)					
Thampi2013	14.5	85.7(6/7)			38.5(5/13)		55.0(11/20)	NA	0.0(0/20)	NA	NA
Gobin2011	24.0	NA	NA	NA	NA	NA	81.7(35/43)	NA	NA	NA	58.4(30/52)
Abramson2016	34.0	NA	NA	NA	85.1(40/47)	NA	78.6(88/112)	0.0(0/112)	2.7(3/112)	NA	72.4(42/58)
Shields2014	19.0	NA	100.0(1/1)	100.0(4/4)	94.1(16/17)	35.7(5/14)	72.2(26/36)	NA	0.0(0/70)	NA	61.8(21/34)
Chen2017	13.6	NA	100.0(11/11)	100.0(11/11)	78.6(44/56)	62.1(18/29)	78.5(84/107)	NA	NA	11.2(12/107)	79.0(49/62)
Tuncer2016	29.0	NA	NA	NA	66.6(16/24)	NA	NA	NA	NA	29.2(7/24)	NA
Shields2011	18.0	NA	NA	100.0(2/2)	100.0(4/4)	33.3(2/6)	66.7(8/12)	0.0(0/16)	0.0(0/16)	18.8(3/16)	50.0(2/4)
Fabian2017	38.7	NA	NA	NA	NA	NA	NA	NA	0.0(0/77)	1.3(1/77)	NA
Munier2017	NA	NA	NA	NA	NA	NA	NA	NA	NA	24.0(6/25)	NA

Note: Globe salvage: avoidance of enucleation or EBRT. Abbreviations: IAC = intra-arterial chemotherapy; NA = not applicable

**Table 3:** Details of trials included in IAC or IVC treatment based on RE grading

Authors	Primary treatment	Mean follow -up (months)	Globe salvage in different group eyes (%)			Overall success rate (%)
			I-III	IV	V	
Gombos2002	IVC	33.0	84.8(28/32)	50(1/2)	62.5(5/8)	81.0(34/42)
Brichard2002	IVC	21.0	100.0(12/12)	NA	57.1(12/21)	72.7(24/33)
Kim2003	IVC	13.0	85.7(12/14)	71.4(5/7)	66.7(4/6)	77.8(21/27)
Abramson2008	IAC	9.0	NA	NA	77.8(7/9)	77.8(7/9)
Abramson2010	IAC	15.0	100.0(2/2)	100.0(1/1)	96.0(24/25)	96.4(27/28)
Gobin2011	IAC	24.0	100.0(8/8)	100.0(4/4)	80.6(29/36)	85.4(41/48)
Shields2011	IAC	24.0	100.0(2/2)	100.0(5/5)	83.3(5/6)	92.3(12/13)

Note: Globe salvage: avoidance of enucleation or EBRT. Abbreviations: IVC = intravenous chemotherapy; IAC = intra-arterial chemotherapy; NA = not applicable

### Primary endpoints

For ICRB grading, the comparison in group A eyes could not be performed due to the absence of data from IAC. Although no significant differences were observed in the globe salvage rates between IAC and IVC in group B eyes (95.8% [95%CI: 57.5%–99.7%] vs. 82.5% [95%CI: 58.9%–94.0%], *P* = 0.163) and group C eyes (91.3% [95%CI: 65.9%–98.3%] vs. 89.0% [95%CI: 69.0%–96.7%], *P* = 0.212), our data suggested that IAC and IVC performed well in terms of globe salvage. The globe salvage rate of IAC was higher than that of IVC in group D eyes (79.5% [95%CI: 71.8%–85.4%] vs. 55.1% [95%CI: 45.6%–64.2%], *P* < 0.001), but not in group E eyes (51.2% [95%CI: 37.0%–65.2%] vs. 43.2% [95%CI: 18.3%–72.1%], *P* = 0.578). Furthermore, the overall success rate was slightly higher in IAC (75.7% [95%CI: 65.7%–83.6%] vs. 69.5% [95%CI: 51.9%–82.8%], *P* < 0.001) than in IVC.

For RE grading, IAC had a higher globe salvage rate than IVC in group IV (90.9% [95%CI: 56.0%–98.7%] vs. 66.3% [95%CI: 32.4%–89.0%], *P* = 0.047) and group V eyes (83.2% [95%CI: 72.0%–90.5%] vs. 59.9% [95%CI: 43.1%–74.6%], *P* = 0.003), but not in group I-III eyes (88.6% [95%CI: 58.3%–97.7%] vs. 88.1% [95%CI: 76.6%–94.4%], *P* = 0.244). The overall success rate was higher in IAC than in IVC (87.1% [95%CI: 78.1%–92.7%] vs. 77.3% [95%CI: 68.1%–84.4%], *P* = 0.033).

### Secondary endpoints

For ICRB grading, five IVC trials (321 eyes) and five IAC trials (249 eyes) reported tumor recurrence. The analysis revealed no difference in the recurrence rate between IAC (15.0% [95%CI: 7.3%–28.4%], P < 0.001) and IVC (15.4% [95%CI: 4.1%–43.8%], *P* = 0.022), with a *P*-value of 0.148. In IVC, metastasis occurred in only one eye [20], whereas it occurred in six eyes in IAC [29, 38]. However, no evidence supported that IVC had an advantage over IAC (2.7% vs. 0.6%, *P* = 0.194). In a study by Turaka et al. [39], 4% (6/156) of patients with germline retinoblastoma treated with IVC as first-line therapy developed SMNs. These SMNs included osteosarcoma (*n* = 3), tectal glioma (*n* = 1), acute promyelocytic leukemia (n = 1), and one patient with both rhabdomyosarcoma (temporal fossa) and conjunctival/orbital malignant melanoma. Shields et al. [25] reported one case of osteosarcoma. However, no SMNs were reported in patients treated with IAC. For RE grading, recurrence occurred in only five eyes (two trials). No tumor metastasis or SMNs was reported.

The details of adverse events of IAC (seven trials) and IVC (nine trials) are listed in Table 4. Adverse events included systemic complications and ocular complications. IAC had more systemic complications than IVC, including neutropenia and infections. Ocular complications were also reported more frequently in IAC, which included vascular injury, spasm, obstruction, and a series of related organ ischemic lesions.

**Table 4:** Adverse events of IVC and IAC in the treatment of retinoblastoma

Trials	Treatment	Patients	Adverse Events
Berry2012	IVC	7	febrile neutropenia
Kaliki2011	IVC	1	pneumonia
Fabian2017	IVC	6	choroidal ischaemia
		4	retinal detachments
		6	nystagmus
Friedman2016	IVC	8	grade 3or 4 toxicity
		2	infections (allergic reaction with urticaria) dehydration)
Yousef2017	IVC	3	rhegmatogenous retinal detachment
Berry2017	IVC	2	febrile neutropenia
Munier2016	IVC	3	occlusive choroidopathy
Thampi2013	IVC	6	infections (fever, acute otitis media, upper) respiratory tract infection)
		3	vitreous hemorrhage
		2	grade 4 neutropenia
		1	cataract
Brichard 2002	IVC	9	non-specific gastrointestinal toxicity
		8	cytopenias
		8	fever
Gobin2011	IAC	6	allergic reaction
		3	cataract
		29	neutropenia
		1	fever
Abramson2016	IAC	39	grade 3 or 4 neutropenia
		5	allergy-type reaction
		4	grade 3 or 4 thrombocytopenia
		4	fever
		6	retinal or choroidal vascular occlusions
		5	phthisis
		4	vitreous hemorrhage
Shields2014	IAC	4	vitreous hemorrhage
		5	artery obstruction
		4	partial choroidal ischemia
Fabian2017	IAC	6	Nystagmus
Abramson2008	IAC	3	lids edema
		3	conjunctiva hyperemia
		1	radiationlike retinopathy
		1	neutropenia
Abramson2010	IAC	9	grade 3 neutropenia
		1	grade 4 neutropenia
Tuncer2016	IAC	9	chorioretinal atrophy
		5	noted retinal detachment
		1	vitreous haemorrhage

IVC: Intravenous Chemotherapy; IAC: Intra-arterial Chemotherapy

### Heterogeneity

For IAC based on ICRB grading, no statistical heterogeneity was found in globe salvage of group B eyes (I^2^ = 0%, *P* = 0.360) and group C eyes (I^2^ = 0%, *P* = 0.765). There was moderate statistical heterogeneity in globe salvage of group D eyes (I^2^ = 26.89%, *P* = 0.242), group E eyes (I^2^ = 40.19%, *P* = 0.188) as well as in the secondary treatment success rate (I^2^ = 44.19%, *P* = 0.127). However, grievous heterogeneity was found in overall success rate (I^2^ = 52.66%, *P* = 0.061) and recurrence rate (I^2^ = 69.44%, *P* = 0.011). For IVC based on ICRB grading, we did not observe significant heterogeneity in globe salvage of group A eyes (I^2^ = 0%, *P* = 0.618) and group C eyes (I^2^ = 0%, *P* = 0.731), but there was grievous heterogeneity in groups B (I^2^ = 77.66%, *P* = 0.004), D (I^2^ = 72.21%, *P* = 0.001), and E eyes (I^2^ = 76.75%, *P* = 0.005). High heterogeneity was also found in overall success rate (I^2^ = 84.20%, *P* = 0.002) and recurrence rate (I^2^ = 92.37%, *P* < 0.001). No statistical heterogeneity was found in all groups of RE grading (I^2^ = 0%, *P* = 0.401–0.924).

## Discussion

The current commonly used treatments for retinoblastoma include IVC and IAC, along with consolidated therapy based on tumor staging. For ICRB grading, we found a significantly higher globe salvage rate for group D eyes in patients who received IAC compared with those who received IVC, while no statistical differences were found in groups A, B, C, and E eyes. IAC was associated with a slightly higher overall success rate than IVC. There was no difference in tumor recurrence and metastasis rates between IAC and IVC. For RE grading, IAC showed a significant advantage in globe salvage of groups IV and V eyes, but not in group I-III eyes. The overall success rate was higher in IAC than IVC.

The aim of this meta-analysis was to quantitatively merge the congeneric rates of included studies that were searched as thoroughly as possible to enlarge the sample size for a more reliable result. To the best of our knowledge, this is the first meta-analysis to compare the outcomes of IVC and IAC for the treatment of retinoblastoma. We made use of multiple statistical rates to compare IVC and IAC across different staging systems to obtain an objective conclusion.

The outcomes of globe salvage of groups A to C eyes may not be reliable because of the unequal or excessively small number of trials. Overall, IAC had significant advantages over IVC in globe salvage of group D eyes and a better overall success rate. However, in approximately half of the group E eyes, treatment with IAC or IVS was unsuccessful, and the eyes eventually had to be removed. To save more diseased eyes at advanced stages, appropriate treatments should be developed. We will focus on this issue in a future study.

There was no significant difference between IAC and IVC regarding tumor recurrence. Extensive recurrence of subretinal or vitreous seeds was the most common cause of treatment failure which led to final enucleation. Shields et al. [11, 30] reported that most recurrences were discovered at the three-year follow-up. In the present study, the follow-up period of patients who received IAC was shorter than three years; therefore, the effect of IAC on tumor recurrence may be over-evaluated.

Some studies have reported adverse events associated with IVC; however, most adverse events would disappear after symptomatic treatment. Rational chemotherapy drug use was essential to reduce the occurrence of adverse events in IVC treatment. In contrast, despite the advantages regarding tumor control, IAC carried a higher risk for potential local complications because of the high concentration of chemical drugs in the eye. The main temporary IAC-related complications that have been reported include eyelid edema, blepharoptosis, forehead hyperemia, and forehead hyperpigmentation, with a mean remission of two weeks to four months [28, 30]. Moreover, IAC-induced vascular events, including vascular injury, spasm, obstruction, and a series of related organ ischemic lesions, deserve attention. Evidence also exists that IAC causes fatal side effects such as stroke or limb ischemia, though they are rare. Most retinal arterial obstructions were found at the one-month follow-up, whereas choroidal vascular atrophy develops slowly and usually takes several months to become apparent [40]. Vascular compromise could lead to poor vision [41], but assessment of vision requires a long follow-up. Therefore, closer visual observation should be recommended for these patients. In addition, the incidence of vascular events can be significantly reduced by precise surgery, careful angiography analysis, and ideal microcatheter placement.

In countries with advanced health care, the incidence of metastasis in children with retinoblastoma is less than 10% [10]. The risk of metastasis greatly increases with histopathologic evidence of high-risk features. Kaliki et al. [22] reported that metastasis did not occur in any patient classified with non-high-risk retinoblastoma, but that death from metastasis occurred in 4% of high-risk patients [42]. Besides, patients with heritable retinoblastoma also have an increased risk of SMNs. The most commonly observed SMNs are sarcomas, melanomas, and myelogenous leukemia, as well as cancers of the nasal cavity, orbit, and brain. The cumulative mortality ratio of an SMNs in patients with heritable retinoblastoma to those with non-hereditable retinoblastoma was 22.5:1 at 50 years of age following a diagnosis of retinoblastoma [13]. As for patients treated with IAC, accumulated exposure to irradiation and the use of melphalan may induce germline mutations and lead to SMNs [43, 44]. Concerning patients treated with IVC, etoposide and carboplatin have been shown to increase the risk of SMNs [45–47]. Moreover, the total dose is also an important factor that should be considered when assessing risk. Most patients with SMNs received higher total doses than patients who received normal treatment. However, in all studies of SMNs, a large proportion of the eyes previously or simultaneously received external radiotherapy. Therefore, the occurrence of SMNs was not necessarily due to chemotherapy; radiation therapy may have been responsible. The effect of IAC and IVC on the prevention of SMNs warrants optimism.

This study had some limitations. Firstly, it was based on data from clinical trials which implied the impossibility of excluding the presence of confounding factors, such as the selection of medications, the severity of the condition (unilateral or bilateral disease, tumor diameter and thickness, subretinal fluid, and subretinal or vitreous tumor seeds), and previous therapies received. In addition, because retinoblastoma is a relatively rare disease, the number of eligible trials was not sufficient for a robust analysis. Moreover, the follow-up time for patients who received IAC was shorter (23.9 months) than for those who received IVC (55.6 months), which could bias the results. It was evident that long-term survival rates gradually declined as the follow-up time extended [19, 25, 30]. Consequently, the results of this meta-analysis were preliminary. Further analyses with more eyes and long-term dynamic observations are necessary to refine the results.

## Conclusions

The results suggested that IAC had advantages in eyes with advanced disease, including groups D, IV, and V, but not in groups A-C, E, and I-III eyes. The overall success rate was higher in IAC than in IVC. However, there were no significant differences between the two methods regarding tumor recurrence and metastasis rates. Larger sample studies with longer follow-up times are warranted to confirm our findings.

## Additional files

Additional file 1: The outcomes of meta-analysis in intra-arterial chemotherapy based on ICRB grading. (DOCX 244 kb)

Additional file 2: The outcomes of meta-analysis in intravenous chemotherapy based on ICRB grading. (DOCX 280 kb)

Additional file 3: The outcomes of meta-analysis in intra-arterial chemotherapy based on RE grading. (DOCX 191 kb)

Additional file 4: The outcomes of meta-analysis in intravenous chemotherapy based on RE grading. (DOCX 174 kb)

## Abbreviations

CI: Confidence interval
EBRT: External beam radiotherapy
IAC: Intra-arterial chemotherapy
ICRB: The International Classification of Intraocular Retinoblastoma
IVC: Intravenous chemotherapy
Mesh: Medical Subject Headings
RE: Reese-Ellsworth
SMNs: Second malignant neoplasms
VEC: Vincristine, etoposide and carboplatin
