# Supplementary material for: Comparison between intravenous chemotherapy and intra-arterial chemotherapy for retinoblastoma: a meta-analysis
Source: BMC Cancer. 2018 Apr 27;18:486. doi: 10.1186/s12885-018-4406-6 (PMC5924469; doi:10.1186/s12885-018-4406-6)

Additional file 2. The outcomes of meta-analysis in intravenous chemotherapy based on ICRB grading


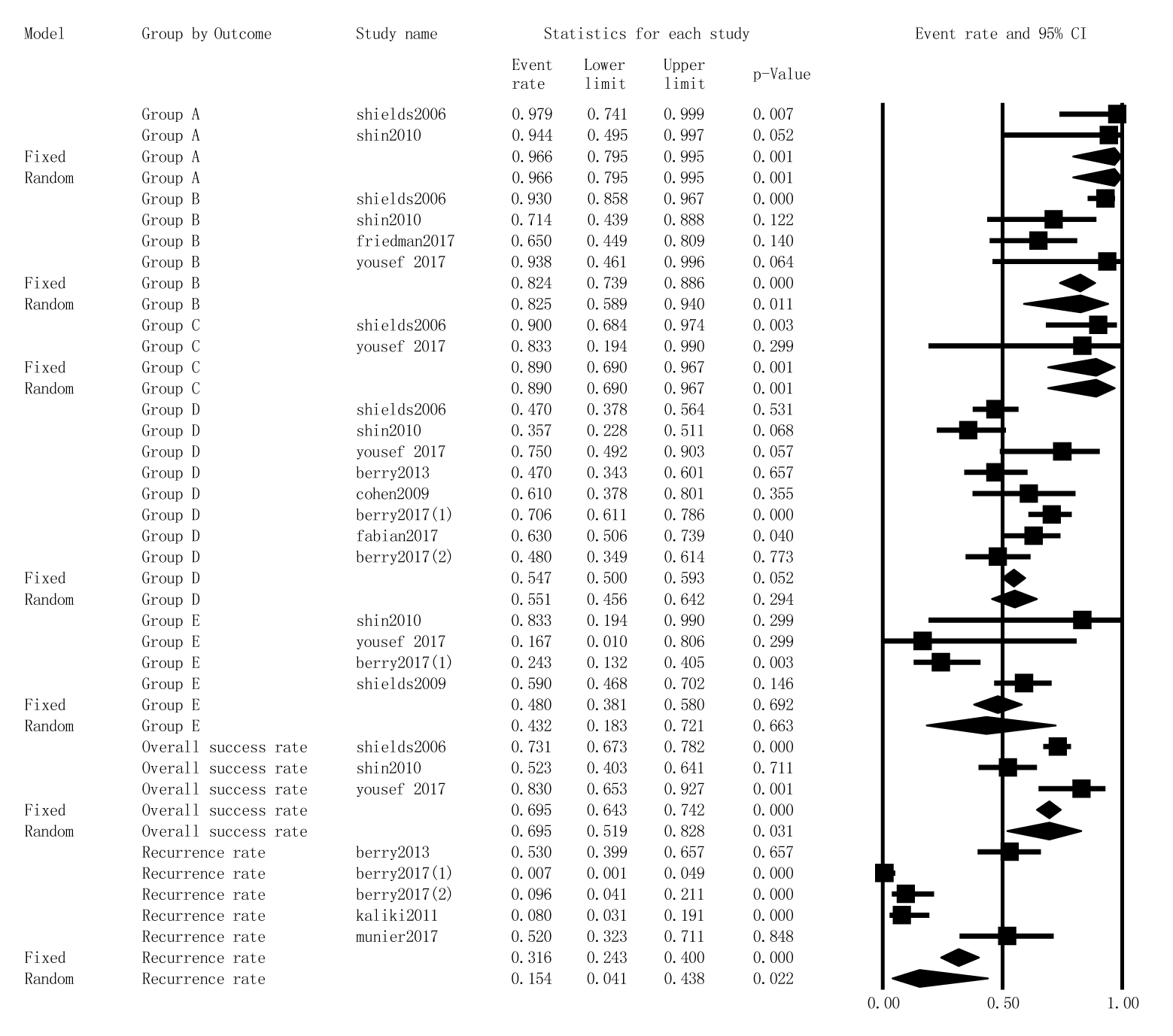

Supplement: Supplementary file 2 — The outcomes of meta-analysis in intravenous chemotherapy based on ICRB grading. (DOCX 280 kb) [file 12885_2018_4406_MOESM2_ESM.docx]
