# Supplementary material for: Comparison between intravenous chemotherapy and intra-arterial chemotherapy for retinoblastoma: a meta-analysis
Source: BMC Cancer. 2018 Apr 27;18:486. doi: 10.1186/s12885-018-4406-6 (PMC5924469; doi:10.1186/s12885-018-4406-6)

Additional file 4. The outcomes of meta-analysis in intravenous chemotherapy based on RE grading


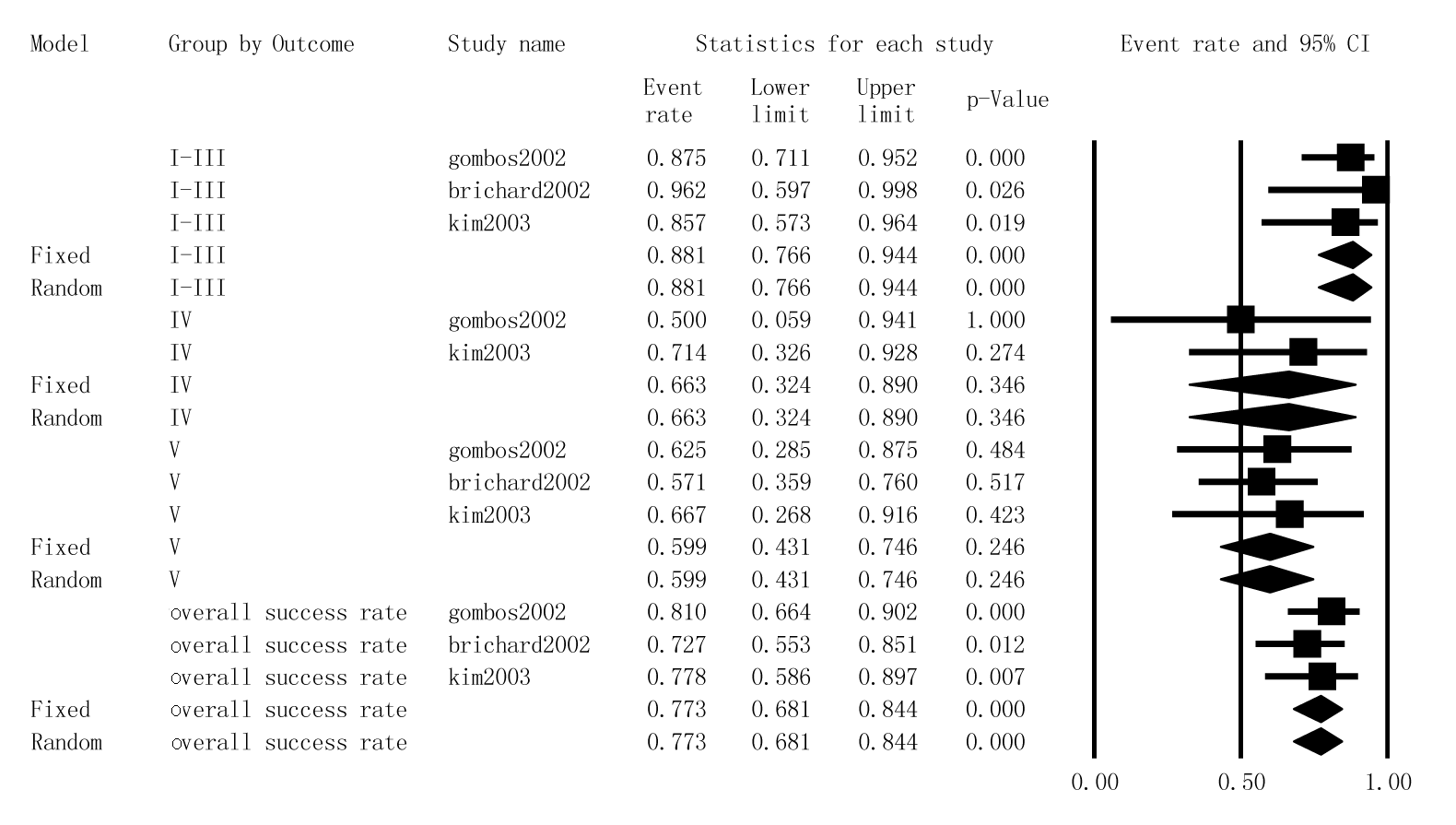

Supplement: Supplementary file 4 — The outcomes of meta-analysis in intravenous chemotherapy based on RE grading. (DOCX 174 kb) [file 12885_2018_4406_MOESM4_ESM.docx]
